# Canadian Medical Degrees in England

**Published:** 1884-02

**Authors:** 


### CANADIAN MEDICAL DEGREES IN ENGLAND.

In a late number of this journal, we called attention to the fact, that while Canadian physicians are able with insignificant restrictions to remove to this country and engage here in the practice of medicine, the Canadian laws regulating such practice in the dominion are such as to practically exclude physicians having American diplomas from similar privilege.

A striking illustration has just been furnished of the position sustained in Great Britain by the " royal " medical institutions of the Dominion.

It seems that a Dr. Chas. Empy, whom our English friends across the water introduce to the public as " Mr." Empy, having been educated in Canada, obtained a degree of M. D. from the Royal College of Physicians and Surgeons, in Kingston, Ontario. He removed to Yorkshire, England, and there became assistant to one Dr. McNab of Crosshills, agreeing not to practice on his own account in that neighborhood. It soon, however, became convenient for the Canadian to cancel or in some way to evade this agreement, so as to begin the practice of medicine and surgery there on his own account.

A jealous Briton was not long in giving alarm to the neighborhood. In a letter to the *Skipton Pioneer*, signed " Veritas," (how often has this specious name been used as a cloak beneath which mortal stabs have been inflicted upon unsuspecting breasts!) it was pointed out that " Mr." Empy's name " is not, and never was, upon the medical register ; and that he had, therefore, no right to assume the title of Doctor nor to practise as a physician and surgeon." " Mr." Empy then began criminal proceedings for libel against the unfortunate *Pioneer*, which resulted, after a prolonged argument before the bench, in the dismissal of the case against the alleged libeller.

In reviewing the points of this case, as they have attracted some attention in Great Britain. it is noticeable that the medical press for the most part take the ground, that " Mr." Empy was simply an unfortunate man. They admit, as indeed they must in the face of the law, that no one can now become entitled to practice medicine in the United Kingdom who has not obtained the requisite degrees in that kingdom.

With the wisdom, the justice, or the necessity of such a law, we are not now concerned. Neither shall we take occasion at this time to express our sympathy with " Mr." Empy, for the hardship which his experience as a practiser in Great Britain has brought to him. It is the " Royal " College of Physicians and Surgeons of Kingston, Canada, for which we mourn. Has it come to this, that that which the Canadians call " royal," is such for the Dominion only ? Is the " royalty " in which our Canadian friends take such keen pride only, after all, a species of pseudo-royalty, which does not hold good in the presence of the genuine article on the other side of the Atlantic ?

If this be so, perhaps it would be as well to change these titles so as to have them read " Royal for Canada Only."

